# A rare paediatric ‘floating elbow’; a supracondylar fracture with an ipsilateral Monteggia fracture: A case report

**DOI:** 10.1016/j.ijscr.2022.107079

**Published:** 2022-04-13

**Authors:** Granit Ismaili, Elsiddig Mahmoud, Pat O' Toole

**Affiliations:** a Children's Health Ireland at Crumlin, Cooley Rd, Crumlin, Dublin D12 N512, Ireland; b Omdurman Islamic University, Khartoum, Sudan

**Keywords:** Monteggia, Floating elbow, Compartment syndrome, Case report

## Abstract

**Introduction and importance:**

A paediatric floating elbow involves fractures of the supracondylar region of the humerus with ipsilateral fracture of the forearm bones. A floating elbow is very uncommon with an incidence of 3 to 13% of all supracondylar fractures. A concomitant supracondylar and Monteggia fracture is extremely rare with only six cases reported in the literature.

**Case presentation:**

We present the unusual case of an eight-year-old girl with a concomitant ipsilateral supracondylar humerus fracture and open Monteggia fracture. Physical examination showed a neurovascularly intact limb. Surgical management was carried out in the form of closed and open reduction, percutaneous pinning using Kirschner (K) wires and Titanium Elastic Nails (TENs), and wound washout and debridement of the open lesion.

The patient developed pin site infection six weeks post operation and subsequently underwent surgery for removal of pins. She was later followed up with normal radiographic and physical examination findings.

**Clinical discussion:**

The complexity of these fractures can lead to debilitating complications if proper management is not initiated. It is imperative that neurovascular and motor function be assessed in great detail and early surgical fixation be carried out in order to prevent these complications.

**Conclusion:**

A paediatric floating elbow is a rare surgical emergency. Although no guidelines for the management of these fractures exist, we recommend surgical management in a step-by-step approach be used over conservative management. We also stress the importance of regular follow up to address any post operative complications that may arise such as the one in our case.

## 1 Introduction

Supracondylar fractures are a common elbow injury in children with an incidence in the literature reported to be between 3.3% and 16.6% [1]. In 1959 JJ Gartland devised a classification scheme for supracondylar lesions based on the degree and direction of displacement, and the presence of intact cortex [2].

Monteggia fractures involve the proximal ulna with an associated radial head dislocation. This was first described as early as 1814 by Italian surgeon Giovanni Monteggia [3]. They account for less than 5% of all paediatric elbow fractures [4]. In 1967 José Louis Bado published a classification scheme of Monteggia lesions based on the direction of the dislocation of the radial head [3]. Bado described four basic types of Monteggia lesions and a group of lesions with similar characteristics, which he termed Monteggia equivalents.

A ‘floating elbow’ is a term coined by Statinski in 1980 to describe a fracture of the supracondylar region with ipsilateral fracture of the forearm bones [5]. When that ipsilateral fracture involves a Monteggia lesion, the incidence is very rare [6].

The complexity of these fractures can lead to debilitating complications if proper management is not initiated. It is imperative that neurovascular and motor function be assessed in great detail.

Early surgical fixation of this lesion and correct management of open wounds as per guidelines has been reported to prevent the above. Many methods are used for the management of the forearm fractures, ranging from closed reduction and casting, to percutaneous fixation to maintain reduction [6]. The purpose of this case is to highlight the complicated nature of these fractures and the importance of appropriate initial examination and surgical management as well as regular follow up.

This work has been reported in line with the SCARE criteria [7].

## 2 Presentation of case

**Fig. 1:**
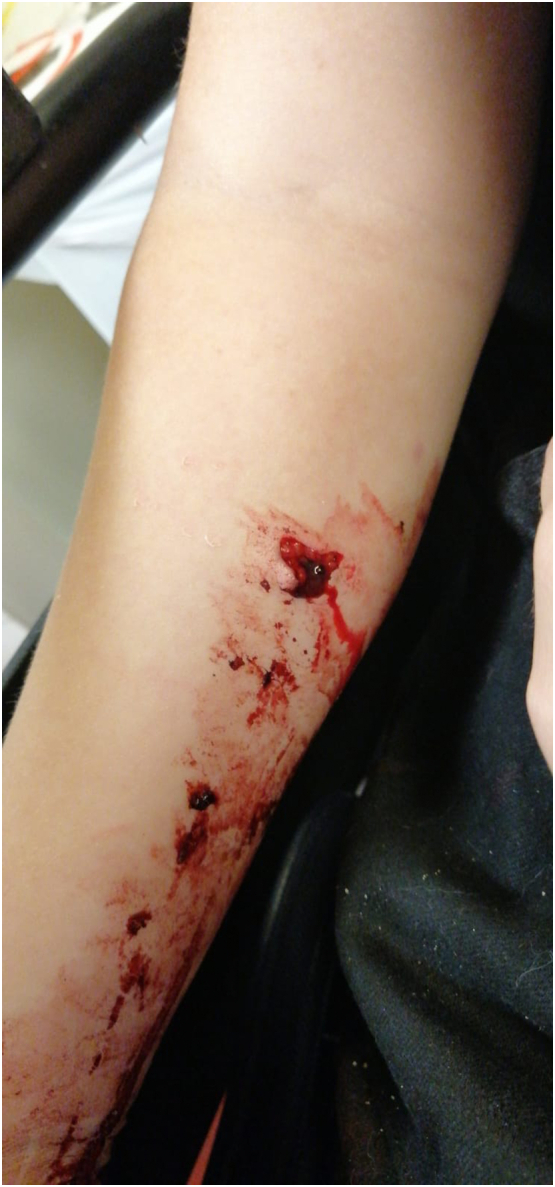
Clinical photograph of the patient in the ED.

**Fig. 2:**
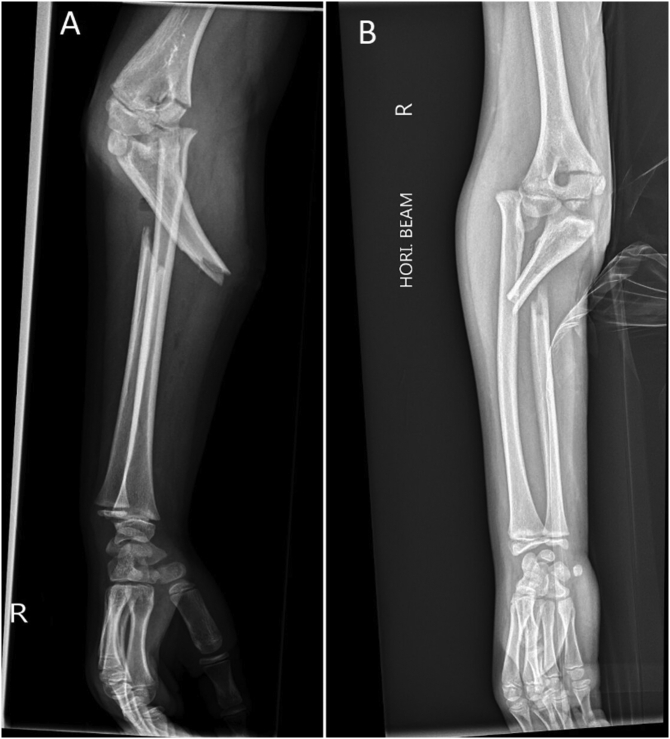
Lateral (A) and anteroposterior (B) views showing a concomitant Monteggia and Supracondylar fracture.

**Fig. 3:**
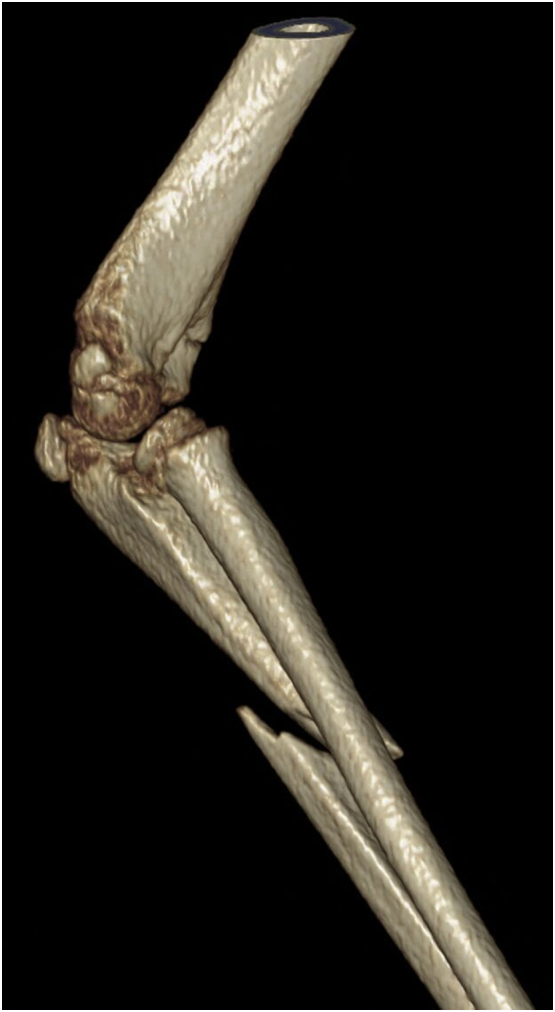
Pre-operative computed tomography image.

An eight-year-old girl presented to the Emergency Department with a right elbow and forearm deformity, and pain after having fallen from a tree at a height of 2 m a few hours prior. This was a low-energy trauma on an out stretched hand. She had no previous medical or family history. Physical examination showed an open proximal forearm volar wound measuring 1.5 cm (Fig. 1). Neurovascular integrity was preserved. X-rays revealed a transverse and minimally displaced supracondylar fracture, a markedly angulated proximal ulnar shaft fracture, a fracture through the radial growth plate (Salter-Harris type 1) and an anterolateral dislocation of the head of the radius (Fig. 2). A back slab was applied post reduction manoeuvre using longitudinal traction with gentle pressure over the proximal radius. A computed tomography scan was performed for preoperative evaluation as we believed this was an unusual injury which needed more thorough imaging (Fig. 3). This was also carried out because arm position for the lateral x-ray was too painful for the child. She was started on intravenous antibiotics and prepared for surgery.

**Fig. 4:**
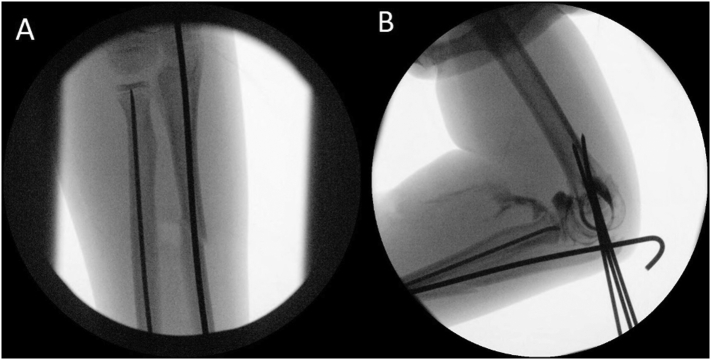
Intraoperative imaging of forearm fixation (A) and arthrogram confirming a reduced radial head (B).

The patient proceeded to theatre that day under general anaesthetic. The operation was performed by the attending consultant with the assistance of two surgical trainees. The open wound underwent copious washout with its edges extended and debrided. The ulnar fracture was reduced and a 2.5 mm anterograde K wire was inserted for fixation. The radial neck fracture was reduced and a 2 mm retrograde TENS nail was applied (Fig. 4). Three lateral 2 mm K-wires were inserted to stabilise the distal humerus fracture. All K-wires were bent and left proud. Lastly an arthrogram was carried out to confirm the correct position of the radial head (Fig. 4). An above elbow back-slab was applied with the forearm in supination. There were no operative complications. The patient was checked for compartment syndrome regularly before being discharged the next day.

**Fig. 5:**
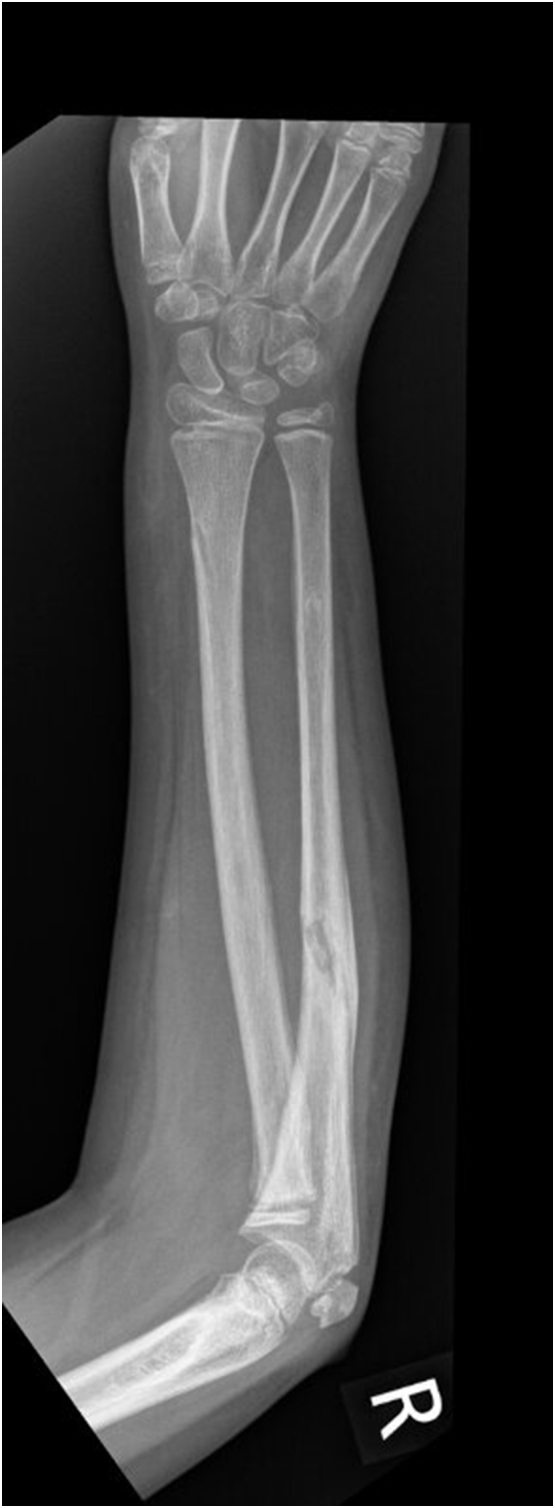
Follow-up x-rays one week after removal of hardware.

After one week the wound was reviewed, a full cast was applied, and an x-ray was performed to show maintenance of reduction (Fig. 5). Repeat imaging after three weeks confirmed satisfactory alignment. The cast was removed after four weeks. Five weeks post-surgery the patient had a fall, landing on both her elbows. She presented to clinic a week later with discharge and erythema at the ulnar pin site. She was taken to theatre the next day for removal of both the K-wires and the TENS nail under general anaesthetic, to prevent further spread of infection. She was kept in hospital for intravenous antibiotics and analgesia for a total of two days. The patient tolerated the surgery quite well, regained full range of motion, and was adherent to post-operative instructions. She engaged well with outpatient physiotherapy and was reviewed clinically up to a year with no further postoperative complications.

## 3 Discussion

Regarding our case, the patient sustained a Gartland type 1 supracondylar lesion and a Bado type 3 Monteggia lesion (antero-lateral radial dislocation). To the best of our knowledge this is the only Bado type 3 lesion presenting with an ipsilateral supracondylar fracture reported in the literature. The associated fracture of the radial head makes our lesion even rarer.

A ‘floating elbow’ is very uncommon with an incidence of 3 to 13% of all supracondylar fractures [8]. A concomitant supracondylar and Monteggia fracture is extremely rare. A review of the literature by Cobanoglue et al. in 2015 found only six cases, including theirs [6].

Due to the scarcity of the literature review, there are no guidelines for the management of these fractures. The treatment methods used in the past have been closed reduction and immobilization with casting by Rouhani et al., Minkowitz et al. and Arora et al. [9], [10], closed reduction with percutaneous pinning by Cobanoglu et al. and Powell et al. [6], [11], and open reduction and internal fixation by Arazi et al. [12]. Similar to our case, Arazi et al. also had an open Monteggia fracture which they fixed with a one 3.5 mm cortical screw [12]. Minkowitz used an arthrogram post manipulation like we did to verify reduction of the radio capitellar joint [10]. Of all the above, Cobanoglu et al. is the only case to have a five year follow up.

Prompt surgical debridement of the open wound was our main priority. We next approached the problem in three steps, dividing each bone with its associated fracture as a separate entity. Taking into consideration the complex nature of the injury we opted for open reduction and K-wiring of the ulna fracture, and intramedullary fixation with TENS for the radius fracture first, followed by percutaneous K-wiring of the supracondylar fracture.

We highly suggest similar surgical intervention as a means of minimizing long lasting complications.

The most common complications from a supracondylar fracture include nerve injury; median nerve (anterior interosseous nerve) at 52% and the radial nerve at 32% [13]. Other complications include vascular insufficiency ranging from 5% to 12% and deformities such as cubitus varus, the most common late complication [14]. Monteggia fracture complications include median and radial nerve injuries, malunion and non-union in 2% to 10% of cases and radial synostosis at 1% to 6% [15]. Compartment syndrome is one of the few true orthopaedic emergencies with the incidence being 7% in ‘floating elbows’ [16]. Clinical examination is paramount in early diagnosis [17]. Our patient underwent repeat serial exams looking for signs of compartment syndrome both before and after surgery.

The preoperative complication our patient faced was having an open fracture. The incidence of open fractures varies from 2% to 9% of all paediatric fractures [18]. We encourage timely administration of appropriate antibiotics before theatre as a means of preventing infection.

Postoperative complications like the pin site infection were dealt with aggressively with surgery booked for the next day and the patient being discharged on oral antibiotics. We recommend sufficient out patient department reviews be based on clinical judgment in order to pick up on such complications.

## 4 Conclusion

Paediatric floating elbows represent a rare surgical emergency. This case provides valuable insight in the correct surgical management of this fracture. It also highlights the importance of pre-operative and post-operative assessment in identifying its many complications. We hope that our case be used as a guide for future managements of this injury.

## 5 Patient perspective

### 5.1 From patient

When I fell out of a tree, from about 2 m high, my twin sister was the only one with me, so she rushed for help. We went straight to Crumlin Hospital from our house. On our way to hospital I felt so much pain I told my Mum I loved her all the way in the car I just wanted to cry.

Check in was too busy so security took us straight to the nurses and they got us help quickly. I got an x-ray and the surgeon was called Dr. O' Toole. There was lots of fuss trying to get an operation done but there was a sick baby in the operation theatre that needed surgery first.

My arm was washed and I got gas so the doctor could push my bones back together but it was still very painful. When the time of surgery came I felt very important, everyone was asking me about the fall. To be honest I felt like a VIP.

Time went by fast, my arm got back to normal. Thanks to all the swimming I did because the physio and the surgeon said I should swim. We went two and sometimes three times a week to the pool and by November I could do everything.

My arm feels and looks 100%.

### 5.2 From mother

My daughters recovery went very well even though we had small setbacks. Overall we were delighted with the results. She has almost full range of motion and she thinks its works and feels 100%.

The displacement of her elbow was frightening along with the open fracture. The team that operated on her did a super job, we are very grateful.

Rehabilitation was two and a half months shorter than expected, she swam a lot. A big thank you to the team that took care of her and made decisions having never seen breaks like Gloria presented with.

## Consent

Written informed consent was obtained from the patient for publication of this case report and accompanying images. A copy of the written consent is available for review by the Editor-in-Chief of this journal on request.

## Provenance and peer review

Not commissioned, externally peer-reviewed.

## Ethical approval

N/a.

## Funding

None.

## Guarantor

2nd author and 3rd author.

## Research registration number

N/a.

## CRediT authorship contribution statement

1st author: wrote most of the manuscript, obtained consent, followed patient during clinical reviews in the outpatient setting
2nd author: conceived the original idea, revised the manuscript, contributed to selection of the images
3rd author: supervised the findings of the work, lead surgeon during the surgery.

## Declaration of competing interest

None.
